# Supplementary material for: Flexible and durable wood-based triboelectric nanogenerators for self-powered sensing in athletic big data analytics
Source: Nat Commun. 2019 Nov 26;10:5147. doi: 10.1038/s41467-019-13166-6 (PMC6879608; doi:10.1038/s41467-019-13166-6)
Supplement: Supplementary file 1 — Supplementary Information [file 41467_2019_13166_MOESM1_ESM.pdf]

Supplementary Information

# Flexible and durable wood based triboelectric nanogenerators for self-powered sensing in athletic big data analytics

Luo et al.

## Supplementary Figures

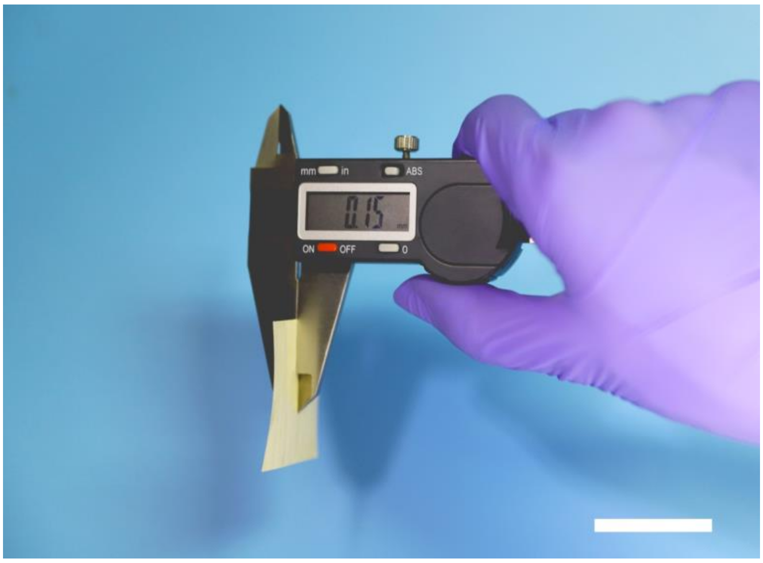


**Supplementary Figure 1. Photograph showing the thickness of the single-electrode mode W-TENG based on treated wood.** Scale bar, 3 cm.

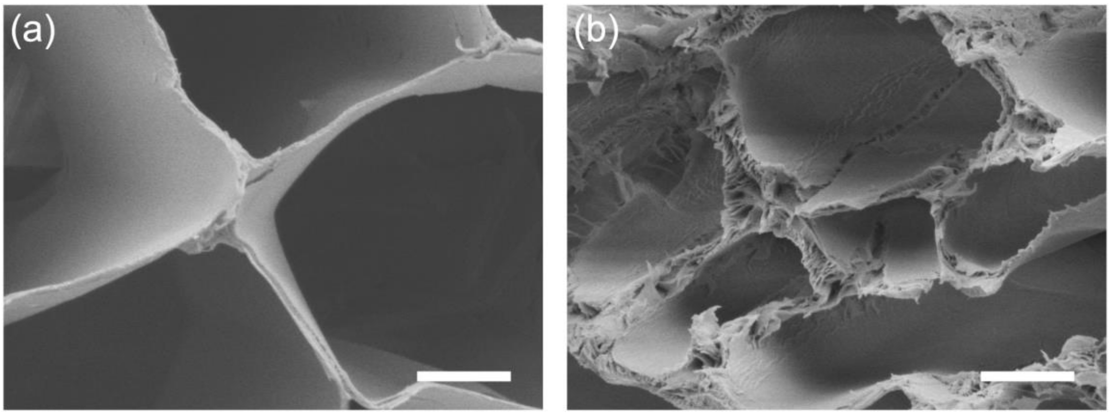


**Supplementary Figure 2.** Enlarged cross-sectional SEM images of the natural wood (a) and treated wood (b). Scale bar, 10 µm.

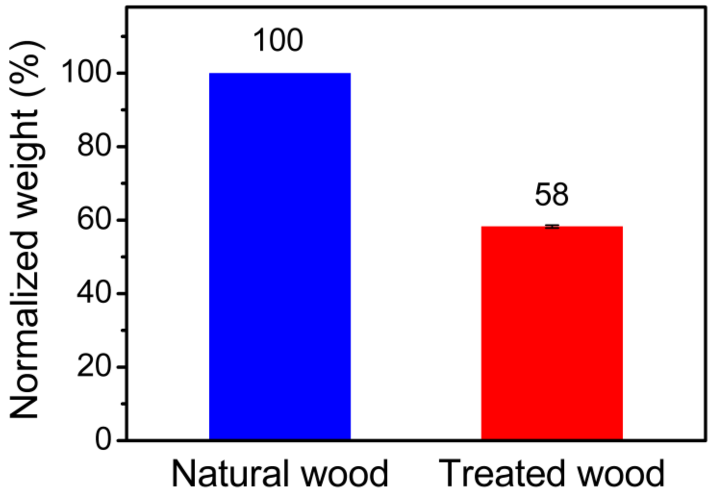


**Supplementary Figure 3. Weight loss of the wood samples before and after chemical treatment.** Error bars indicate standard deviations for 3 sets of data points.

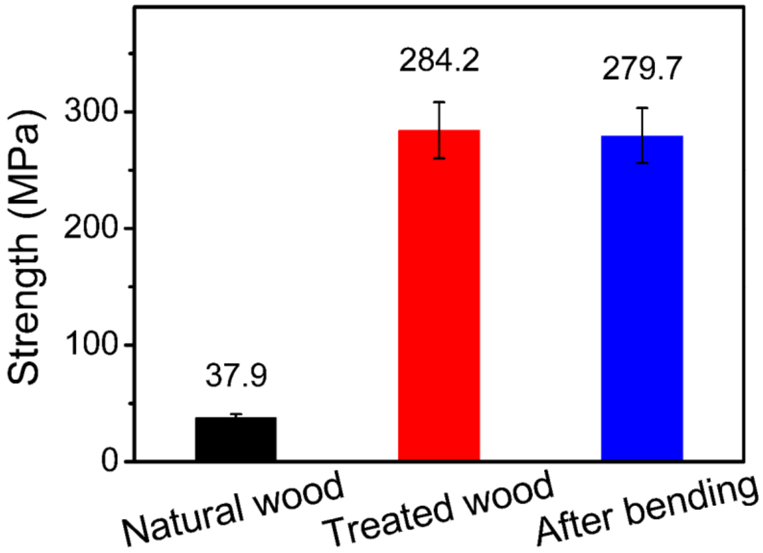


**Supplementary Figure 4. Compared with the natural wood (37.9 ± 3.0 MPa), the treated wood (284.2 ± 24.1 MPa) has greatly improved strength (7.5 times) even after bending 1000 cycles (279.7 ± 23.6 MPa).**

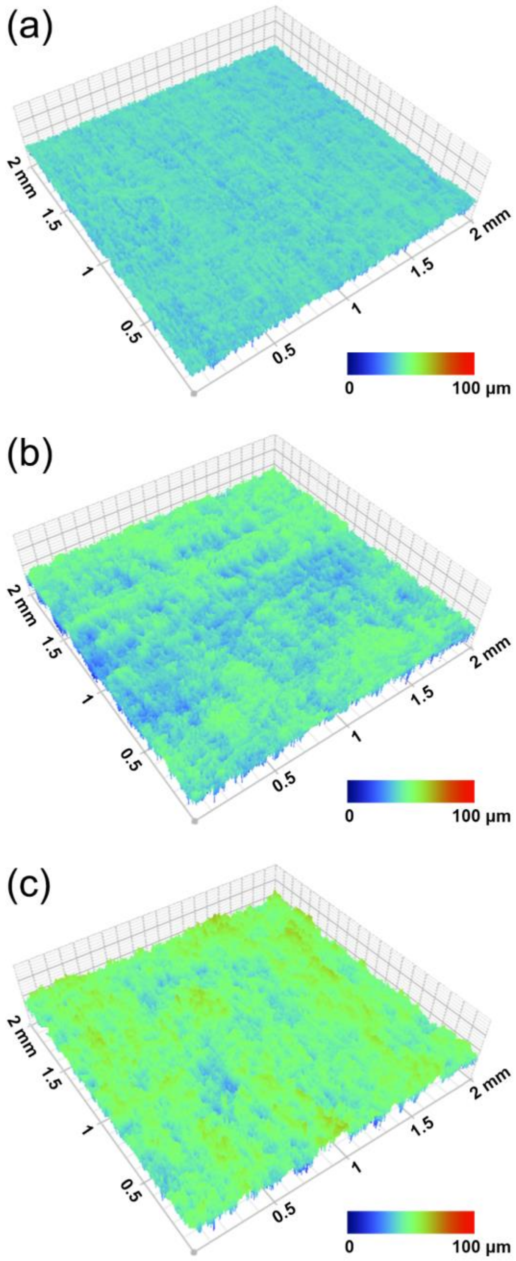


**Supplementary Figure 5. Interferometer images showing the surface topography of the treated wood after hot pressing using various sand papers as the mold.** (a) control. (b) 2000-grit. (c) 1000-grit.

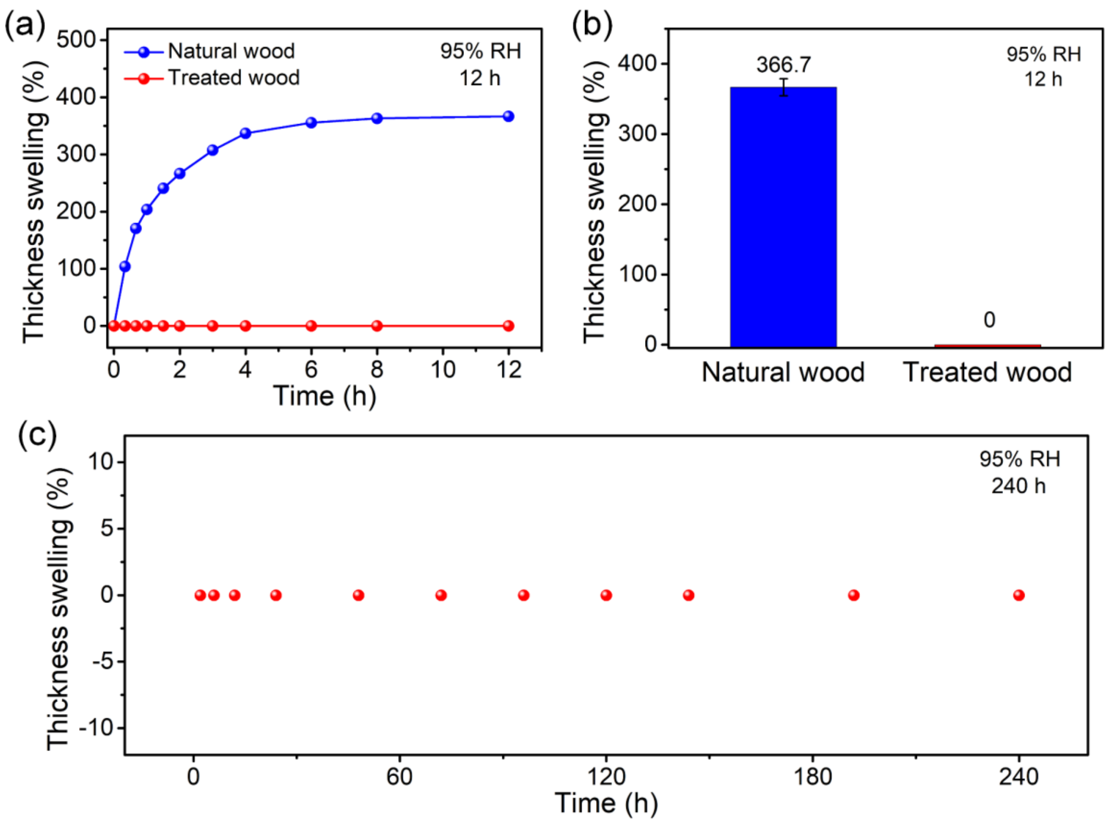


**Supplementary Figure 6. Dimensional stability of the pressed natural wood and treated wood against moisture.** (a) Change in thickness of the pressed natural wood and treated wood under 95% RH over time. (b) Percentage increase in thickness of the pressed natural wood and treated wood after sustaining 95% RH for 12 hours. (c) Long-term change in thickness of the treated wood under 95% RH for 240 hours.

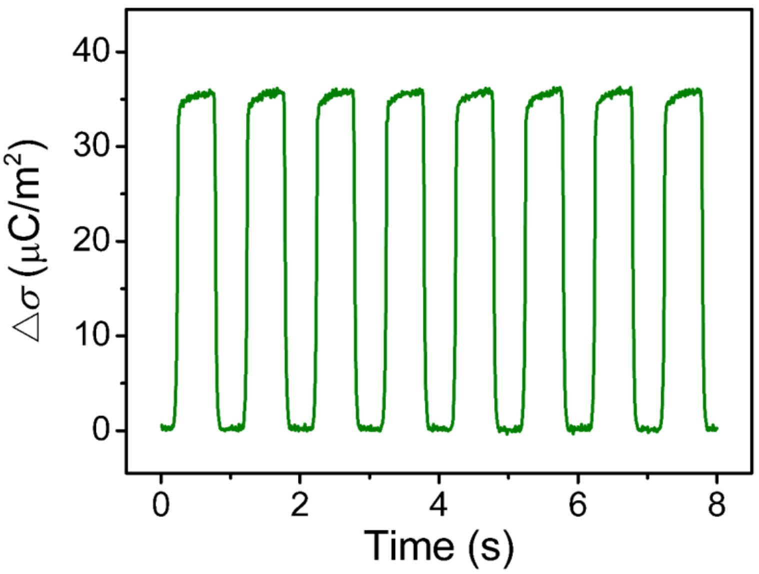


**Supplementary Figure 7. Transferred charge density of the W-TENG based on treated wood in single-electrode mode.**

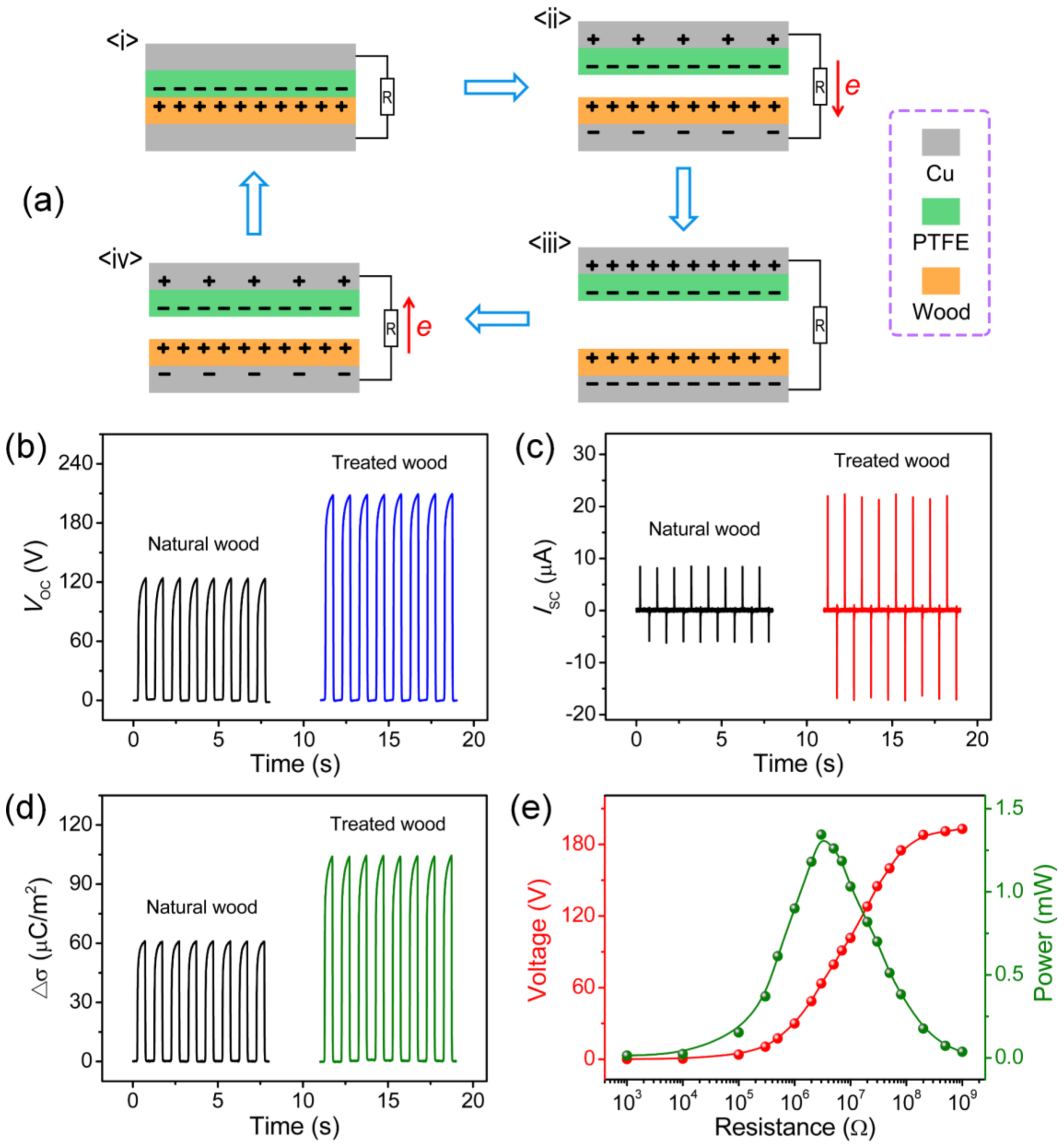


**Supplementary Figure 8. Working mechanism and output performance of the contact-separation mode W-TENG.** (a) Schematics of the operating principle for the W-TENG. (b-d) Open-circuit voltage, short-circuit current and transferred charge density of the W-TENG based on natural wood and treated wood. (e) Dependence of the output voltage and peak power of the W-TENG on the resistance of external loads.

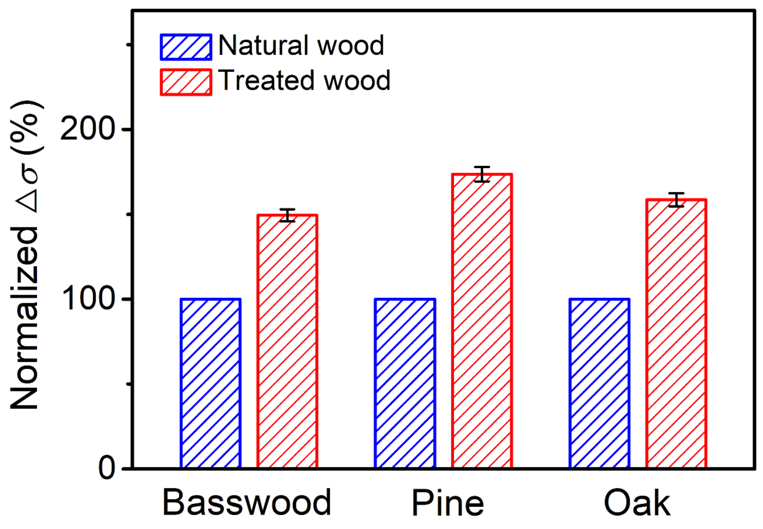


**Supplementary Figure 9. Comparison of the transferred charge density between W-TENGs based on various species of natural wood and treated wood.** Error bars indicate standard deviations for 3 sets of data points.

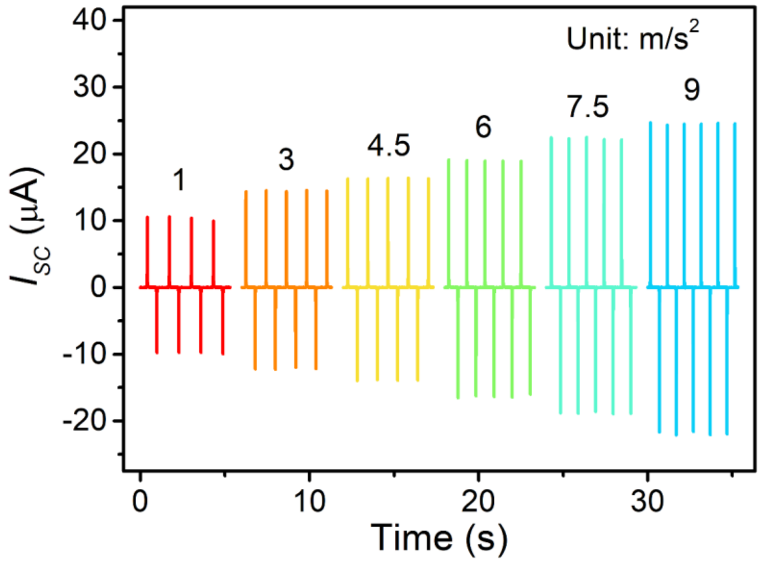


**Supplementary Figure 10. Output current of the W-TENG based on treated wood with different motion accelerations.**

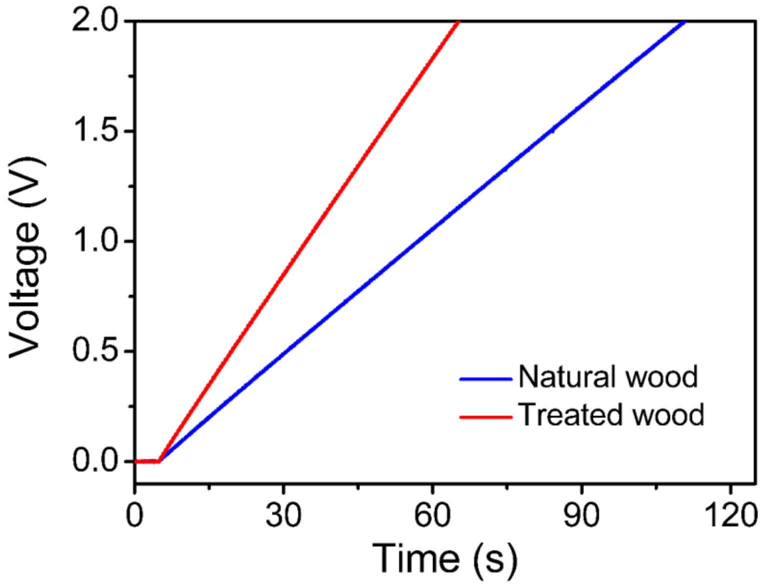


**Supplementary Figure 11. Measured voltage of a 4.7 μF capacitor charged by the W-TENG based on natural wood and treated wood at an agitation frequency of 3 Hz.**

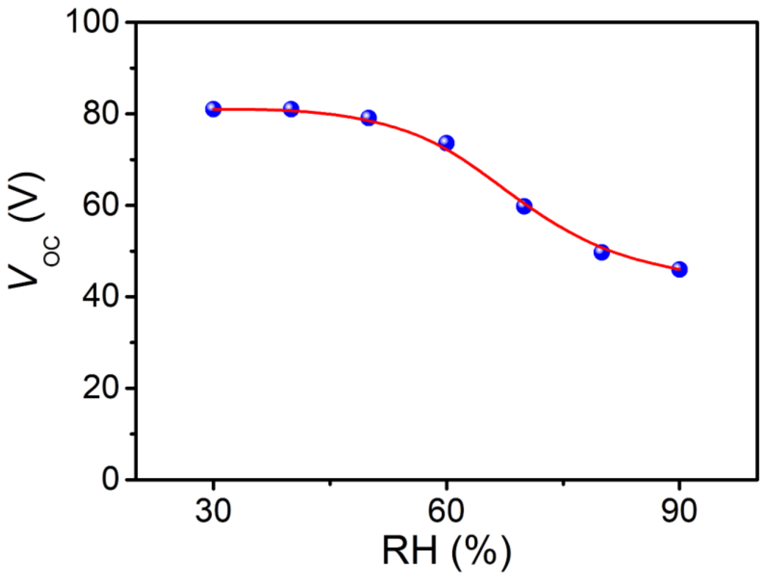


**Supplementary Figure 12. Dependence of the open-circuit voltage of the W-TENG on RH.**

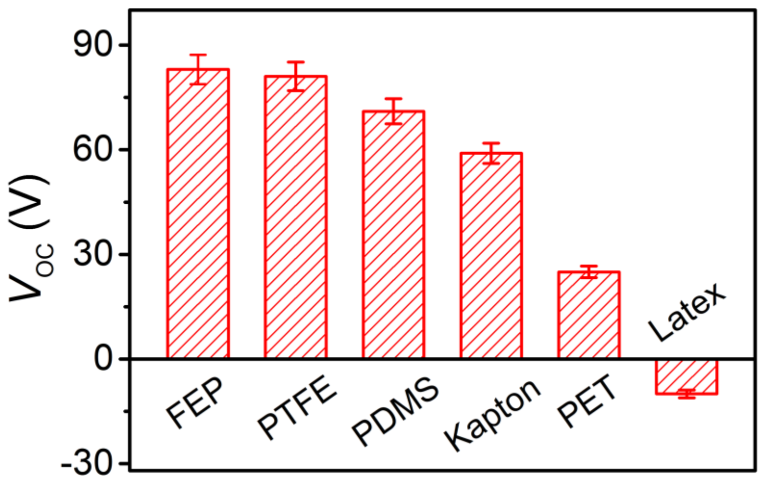


**Supplementary Figure 13. Summarized open-circuit voltage of a W-TENG with relative contact-separation motion to different materials.** The output depends on the relative ability of a dielectric material to gain electrons when contacting with the treated wood, coincident with the well-established tribo-series table[1,2]. Error bars indicate standard deviations for 3 sets of data points.

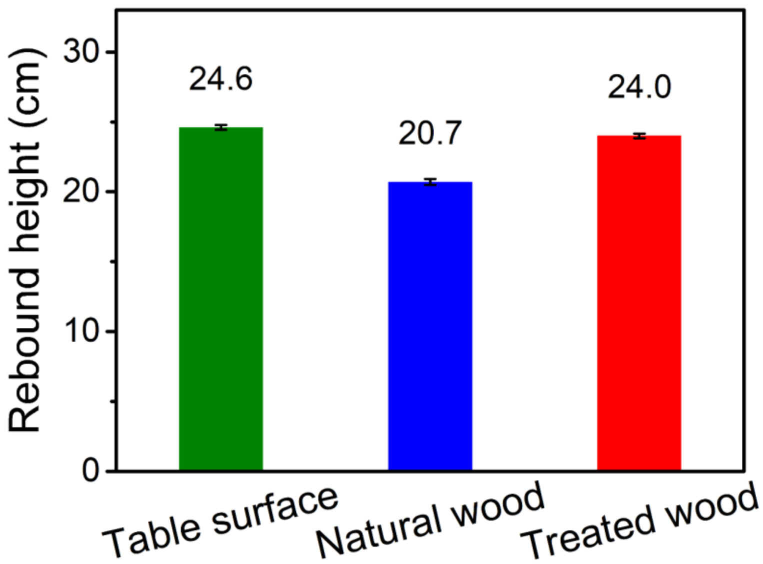


**Supplementary Figure 14. Influence on the elastic property of the table surface by the W-TENG based on natural wood and treated wood.** The rebound height is measured by dropping a standard ping-pong ball onto the surface of the table. Error bars indicate standard deviations for 3 sets of data points.

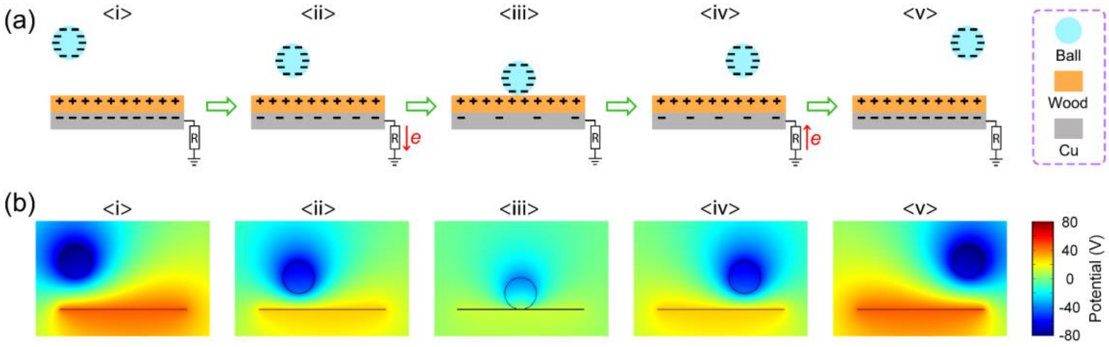


**Supplementary Figure 15.** (a) Schematics of working principle for the ping-pong ball impacting on the single-electrode mode W-TENG. (b) Potential simulation by COMSOL to elucidate the working principle.

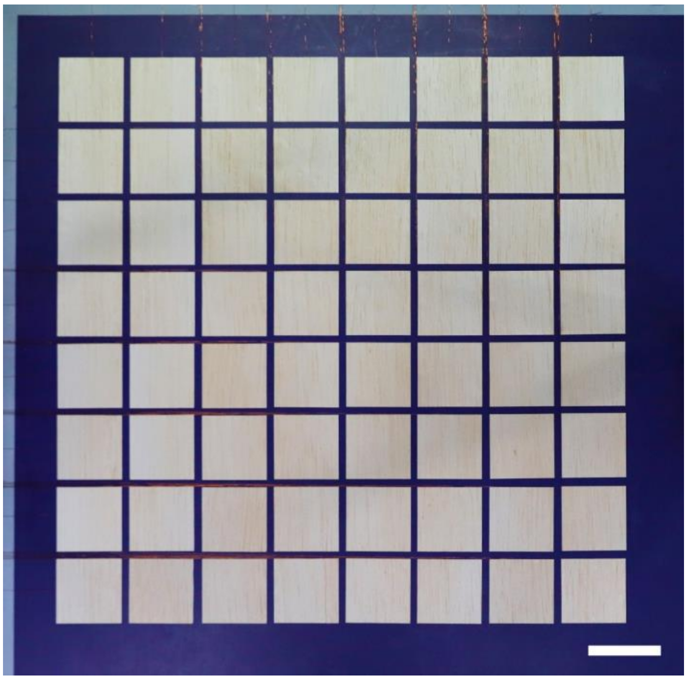

**Supplementary Figure 16. Photograph of the 8 × 8 W-TENG array**. Scale bar, 5 cm.

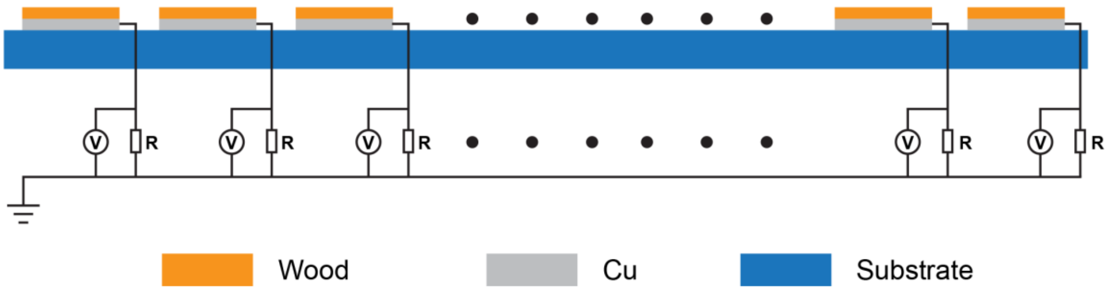


**Supplementary Figure 17. Schematic diagram showing the working principle of the multi-channel measurement system for the smart ping-pong table.**

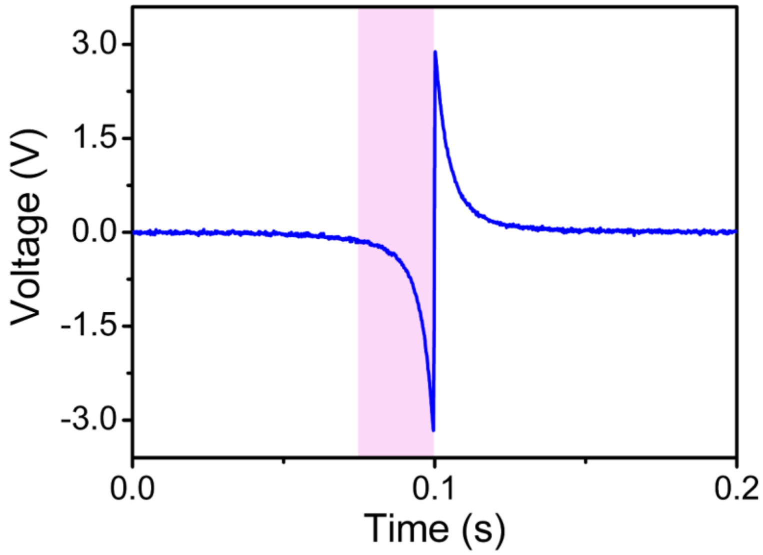


**Supplementary Figure 18. Real-time fast response (25 ms) of the W-TENG as a self-powered velocity sensor.**

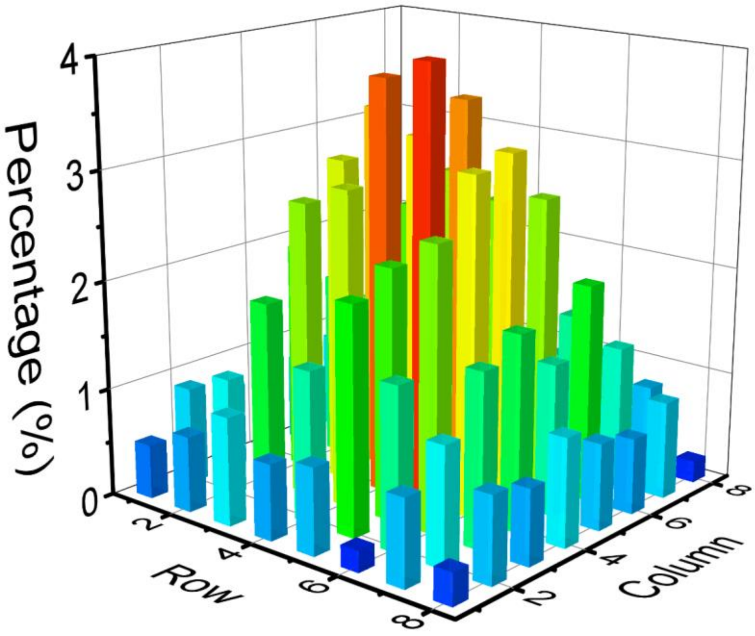


**Supplementary Figure 19. 3D bar chart showing the distribution of statistical percentage of the falling points.**

**Supplementary Notes**

**Supplementary Note1. Working mechanism and output performance of the contact-separation mode W-TENG.**

To investigate the enhancement of the triboelectric property of the wood after treatment, output performance of the W-TENG based on natural contact-separation mode are compared, which is more reliable and representative for performance comparison[3,4]. Different from the single-electrode mode, another Cu electrode is attached at the back of the PTFE film and connected to the bottom Cu electrode through the external circuit (Supplementary Figure 7a). The open-circuit voltage ($V_{OC}$), short-circuit current ($I_{SC}$), and transferred charge density ($\triangle\sigma$) of the W-TENG based on natural wood are shown in Supplementary Figure 7b-d, with peaks of 124 V, 8.5 μA, and 55 μC/m$^2$, respectively. When using the treated wood for fabricating TENGs, its $V_{OC}$, $I_{SC}$, and $\triangle\sigma$ can be improved to 209 V, 22.4 μA, and 94 μC/m$^2$, under the same motion acceleration of 7.5 m/s$^2$. By comparing their output charges, TENGs based on treated wood is 71% higher than that of the natural wood. To investigate the effective output performance of the W-TENG based on treated wood, the output voltage was measured with various resistances applied as the external load. The maximum peak output power of the W-TENG based on treated wood reaches 1.34 mW under an external load resistance of 3 MΩ , as shown in Supplementary Figure 7e.

**Supplementary Note2. Approaching velocities detection of the ping-pong ball.**

The ping-pong ball was free falling from various heights to achieve different approaching velocities. And these velocities were calculated via the following expression without taking the effect of air damping into consideration:

$$v = \sqrt{2gh} \quad (1)$$

In this equation, $v$ represents the approaching velocity, $g$ is the local gravitational acceleration, and $h$ is the falling height.

**Supplementary References**


1 Davies, D. K. Charge generation on dielectric surfaces. *J. Phys. D Appl. Phys.* **2**, 1533-1537 (1969).

2 Diaz, A. F., & Felix-Navarro, R. M. A semi-quantitative tribo-electric series for polymeric materials: the influence of chemical structure and properties. *J. Electrostat.* **62**, 277-290 (2004).

3 Zi, Y. et al. Standards and figure-of-merits for quantifying the performance of triboelectric nanogenerators. *Nat. Commun.* **6**, 8376 (2015).

4 Zou, H. et al. Quantifying the triboelectric series. *Nat. Commun.* **10**, 1427 (2019)
