# Supplementary material for: Flexible and durable wood-based triboelectric nanogenerators for self-powered sensing in athletic big data analytics
Source: Nat Commun. 2019 Nov 26;10:5147. doi: 10.1038/s41467-019-13166-6 (PMC6879608; doi:10.1038/s41467-019-13166-6)
Supplement: Supplementary file 3 — Description of Additional Supplementary Files [file 41467_2019_13166_MOESM3_ESM.pdf]

Description of Additional Supplementary Files

File Name: Supplementary Movie 1
Description: Illustration of the self-powered falling point distribution statistical system

File Name: Supplementary Movie 2
Description: Demonstration of the self-powered falling point distribution statistical system

File Name: Supplementary Movie 3
Description: High speed camera view of the top edge ball

File Name: Supplementary Movie 4
Description: High speed camera view of the side edge ball

File Name: Supplementary Movie 5
Description: Self-powered edge ball judgement system
